# Maternal body mass index and child haemoglobin: A national cross-sectional study in Peru

**DOI:** 10.1371/journal.pone.0359236

**Published:** 2026-09-25

**Authors:** Arnold F. Julca Avalos, Jeniffer K. Pastor Aponte, Victor A. Ramos Berrocal, David R. Soriano-Moreno

**Affiliations:** School of Medicine, Faculty of Health Sciences, Universidad Peruana Unión, Lima, Peru; Universidade de Sao Paulo Faculdade de Saude Publica, BRAZIL

## Abstract

**Background:**

Childhood anaemia remains a major public health problem in Peru. Although several maternal and environmental factors have been implicated, the association between maternal body mass index (BMI) and anaemia in young children remains inconsistent. This study aimed to evaluate the association between maternal BMI and both anaemia and haemoglobin levels in children aged 6–59 months in Peru.

**Methods:**

We conducted a cross-sectional analytical study using data from the 2024 Peruvian Demographic and Family Health Survey. Children aged 6–59 months and their mothers were included. Maternal BMI was the exposure variable, while childhood anaemia and haemoglobin concentration were the outcomes. Poisson regression was used to estimate prevalence ratios for anaemia, and linear regression models were applied to assess associations with haemoglobin levels.

**Results:**

A total of 16,973 children were analysed. In adjusted analyses, maternal BMI categories were not associated with anaemia prevalence. However, maternal obesity, compared to normal weight, was associated with higher haemoglobin levels in children (adjusted β 0.72 g/dL; 95% CI 0.23 to 1.22). Additionally, each 1 kg/m^2^ increase in maternal BMI was associated with a 0.07 g/dL increase in child haemoglobin (adjusted β 0.07; 95% CI 0.03 to 0.11).

**Conclusion:**

Maternal body mass index was not associated with the prevalence of anaemia, although a positive association was observed with child haemoglobin levels. These findings support the inclusion of maternal nutritional status within comprehensive anaemia prevention strategies and highlight the need for longitudinal studies to confirm these associations.

## Introduction

Anaemia remains a public health problem worldwide, affecting approximately 41.4% of children under five years of age, with iron deficiency being the main cause [1,2]. This condition accounts for 1.8 million deaths annually and 1,252 years lived with disability per 100,000 children under five years, more frequently affecting males and children from low-income countries [3,4]. In Peru, in 2024, according to the Demographic and Family Health Survey (ENDES), the prevalence of anaemia in children under five years was 33.7%, reaching 43.7% among children under three years of age [5]. This condition increases the risk of stunting and impairments in cognitive and psychomotor development, particularly during the early years of life [6,7].

Various factors have been associated with childhood anaemia, including type of diet, gestational anaemia, socioeconomic conditions, maternal educational level and area of residence [8,9]. In addition, systematic reviews consistently show that poorer maternal nutritional status is associated with poorer nutritional status in the infant [10,11]; however, the influence of maternal nutritional status on childhood anaemia has been less explored. On the one hand, in underweight mothers, a low body mass index (BMI) may reflect household food insecurity or diets of low nutritional quality, with limited consumption of foods rich in bioavailable iron [12,13]. In contrast, in mothers with overweight or obesity, a higher BMI could act as a marker of greater food availability and access to resources, which could potentially be associated with higher iron intake and higher haemoglobin levels in the child. However, it is also possible that hypercaloric diets that are poor in micronutrients may not ensure adequate iron intake, which could contribute to low haemoglobin levels [10,14,15]. Furthermore, the presence of maternal eating disorders, low nutritional education or permissive or restrictive feeding styles may modulate iron availability in the infant’s diet.

Previous studies conducted in lower-middle-income countries among children under five years have shown that mothers with higher BMI tend to have children with higher haemoglobin levels, whereas maternal underweight is associated with lower haemoglobin [16–18]. In Peru, a high prevalence of the double burden of malnutrition has been described, where maternal overweight or obesity coexists with childhood anaemia [19]. This suggests a possible interrelationship between maternal nutritional status and the child’s haematological profile; however, this association has not yet been evaluated at the national level using representative data. Given the inconsistency of previous evidence and the coexistence of multiple forms of malnutrition in the country, it is necessary to generate evidence to determine whether maternal BMI constitutes a factor associated with childhood anaemia and haemoglobin levels. Understanding this relationship is relevant for the design of comprehensive interventions that address not only child supplementation but also maternal nutritional status and household feeding dynamics to reduce the burden of anaemia in Peru.

Therefore, the aim of this study was to evaluate the association between maternal BMI and anaemia and haemoglobin levels in children aged 6–59 months in Peru.

## Methods

### Design and population

This was a cross-sectional analytical study using data from the ENDES 2024. The study followed the Strengthening the Reporting of Observational Studies in Epidemiology (STROBE) guidelines. ENDES is a survey conducted by the National Institute of Statistics and Informatics of Peru (INEI) and is carried out annually, providing nationally representative data of the Peruvian population. It uses a probabilistic, stratified and multistage sampling design. In 2024, a total of 36,760 households were scheduled to be surveyed, as specified in the ENDES 2024 technical report [20]. For the present study, children aged 6–59 months and their mothers with complete data on the variables of interest were included. Pregnant women were excluded.

### Variables

The dependent variable was anaemia and haemoglobin among children aged 6–59 months, obtained from the Individual questionnaire of ENDES. Altitude-adjusted haemoglobin levels in g/dL and anaemia status according to the updated 2024 technical guidelines of the Peruvian Ministry of Health and the World Health Organization (WHO) were used. Haemoglobin was measured in ENDES using a portable HemoCue haemoglobinometer [20]. Anaemia classification was performed according to age group. Among children aged 6–23 months, severe anaemia was defined as haemoglobin <7.0 g/dL, moderate as 7.0–9.4 g/dL and mild as 9.5–10.4 g/dL [21]. Among children aged 24–59 months, severe anaemia was defined as <7.0 g/dL, moderate as 7.0–9.9 g/dL and mild as 10.0–10.9 g/dL [21].

The independent variable was BMI among women of reproductive age, obtained from the Individual questionnaire. BMI was categorised according to WHO cut-off points as underweight (<18.5 kg/m^2^), normal weight (18.5–24.9 kg/m^2^), overweight (25.0–29.9 kg/m^2^) and obesity (≥30 kg/m^2^) [22].

Additional covariates were evaluated. Contextual and household characteristics included area of residence, natural region, altitude above sea level, household wealth index and quality of drinking water. Child characteristics included age in months, sex, presence of diarrhoea, cough and fever in the previous two weeks, and antiparasitic medication use in the past 12 months. Maternal characteristics included age, marital status, educational level and parity.

### Statistical analysis

Statistical analyses were performed using STATA version 19.0. Databases from the Household and Individual questionnaires were merged using unique identifiers. These databases are publicly available on the INEI website [23]. Data cleaning was conducted by excluding participants with missing data in the variables of interest or implausible values. Missing data were handled according to a pre-specified strategy based on the proportion of missing values for each variable: variables with less than 10% missing data were retained in the analysis, those with 10–30% missing data were considered for multiple imputation, and those with more than 30% missing data were excluded. None of the variables met the criteria for multiple imputation; therefore, no multiple imputation was performed. Accordingly, variables related to iron supplementation, type of breastfeeding received and prematurity had a high proportion of missing values (61.9%, 41.9%, and 91.9%, respectively) and were therefore not included in the analysis. All analyses accounted for the complex survey design using the svy command. Categorical variables were presented as proportions with 95% confidence intervals (95% CI), and continuous variables were described as means with 95% CI. To assess the association between maternal BMI and anaemia in children aged 6–59 months, Poisson regression models were used to estimate crude and adjusted prevalence ratios (PR) with 95% CI. To evaluate the association between maternal BMI and haemoglobin levels (g/dL), linear regression models were applied to estimate crude and adjusted beta coefficients with 95% CI. Multicollinearity was assessed and was not present (variance inflation factor <10). The adjusted model followed an epidemiological approach, including confounders and neutral control variables based on theoretical considerations [24]. A p-value <0.05 was considered statistically significant.

### Ethical considerations

This study was conducted using publicly available survey data that ensure participant confidentiality and anonymity. ENDES obtains informed consent from respondents and complies with ethical principles for research involving human subjects. Additionally, the study protocol was approved by the Research Ethics Committee of Universidad Peruana Unión.

## Results

**Fig 1:**
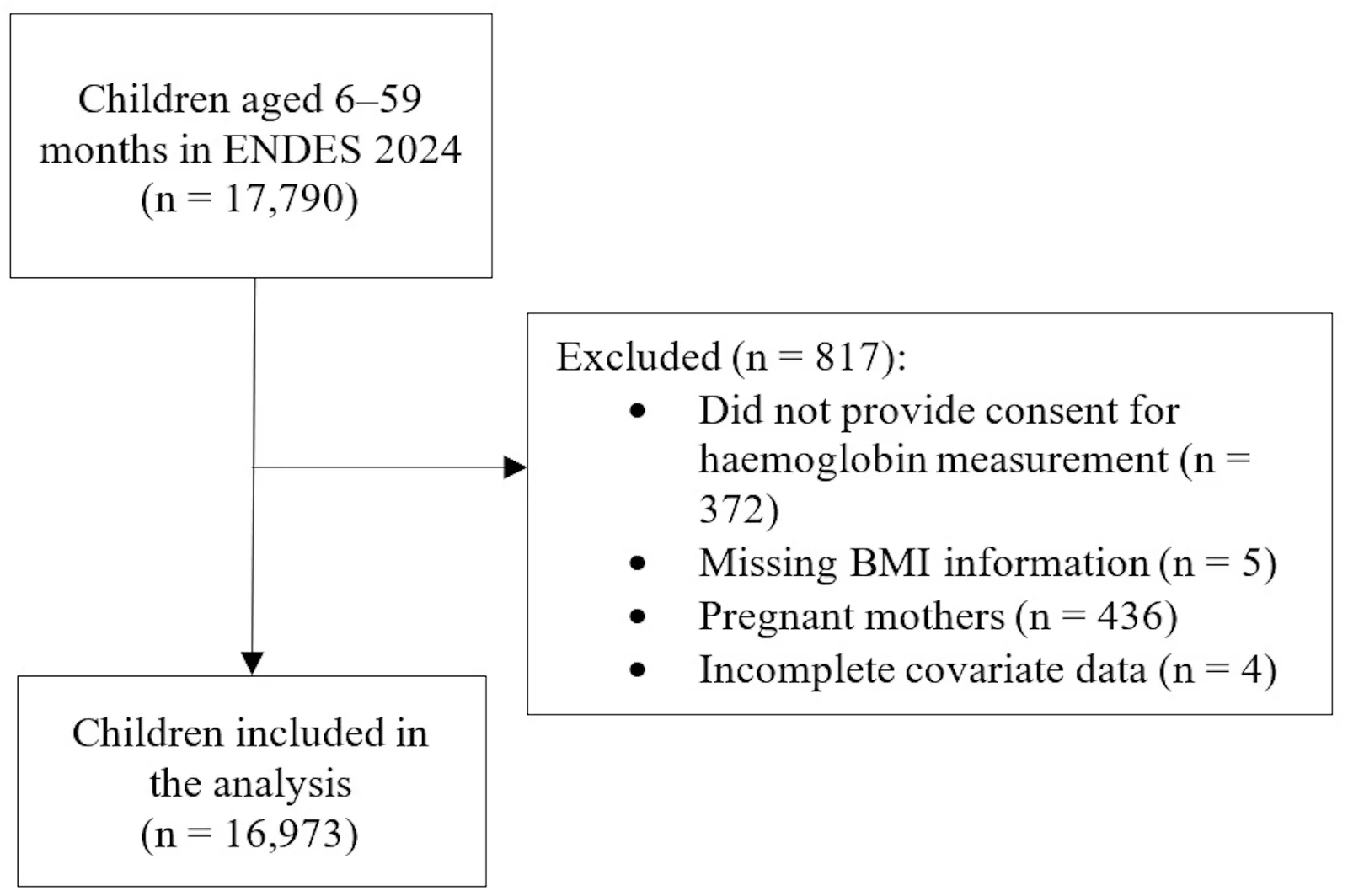
Flow diagram of participant selection.

Initially, 17,790 children aged 6–59 months were identified in the ENDES 2024 database, of whom 16,973 were ultimately included in the analysis (Fig 1).

The mean age of the children was 32.2 months and 51.0% were male. Regarding maternal characteristics, the mean maternal age was 31.1 years. Most mothers resided in urban areas (71.0%), were married or cohabiting (82.2%), had secondary education (47.5%), and had two to three children (54.5%). Concerning child morbidity in the previous two weeks, 34.9% had cough, 20.2% had fever and 12.6% had diarrhoea. The mean maternal BMI was 27.9 kg/m^2^, and most mothers had overweight (40.1%). The mean haemoglobin concentration among children was 11.2 g/dL, and 29.9% had anaemia (Table 1).

**Table 1:** Sociodemographic and clinical characteristics of children aged 6–59 months and their mothers (n = 16,973).

Variables	% or mean (95% CI)
Child age (months), mean	32.2 (32.0 to 32.5)
Maternal age (years), mean	31.1 (31.0 to 31.2)
Child sex	
Male	51.0 (50.1 to 52.0)
Female	49.0 (48.0 to 49.9)
Area of residence	
Urban	71.0 (70.2 to 71.9)
Rural	29.0 (28.1 to 29.8)
Marital status	
Married/cohabiting	82.2 (81.5 to 83.0)
Never married/not living together	17.5 (16.8 to 18.3)
Widowed/divorced	0.3 (0.2 to 0.4)
Natural region	
Metropolitan Lima	22.7 (21.8 to 23.5)
Rest of the Coast	28.0 (26.9 to 29.1)
Highlands	28.9 (27.6 to 30.3)
Jungle	20.4 (19.4 to 21.5)
Altitude (metres above sea level), mean	1131.6 (1095.1 to 1168.1)
Educational level	
No formal education	0.9 (0.8 to 1.2)
Primary	15.6 (14.8 to 16.5)
Secondary	47.5 (46.4 to 48.6)
Higher	36.0 (34.9 to 37.0)
Wealth index	
Poorest	28.9 (27.9 to 30.0)
Poor	24.1 (23.1 to 25.1)
Middle	19.4 (18.6 to 20.3)
Rich	15.8 (15.0 to 16.7)
Richest	11.7 (11.0 to 12.5)
Parity	
One child	27.8 (26.9 to 28.6)
Two to three children	54.5 (53.5 to 55.5)
Four or more children	17.7 (16.9 to 18.5)
Water quality	
Safe water	36.9 (35.7 to 38.1)
Partially chlorinated	11.1 (10.3 to 11.8)
Unsafe/non-chlorinated	39.4 (38.3 to 40.7)
Not measured	12.6 (11.8 to 13.4)
Diarrhoea (past 2 weeks)	
No	87.4 (86.8 to 88.0)
Yes	12.6 (12.0 to 13.2)
Cough (past 2 weeks)	
No	65.1 (64.0 to 66.1)
Yes	34.9 (33.9 to 36.0)
Fever (past 2 weeks)	
No	79.8 (79.0 to 80.6)
Yes	20.2 (19.4 to 21.0)
Antiparasitic medication (past 12 months)	
No	59.9 (58.9 to 60.8)
Yes	40.1 (39.2 to 41.1)
Maternal BMI (kg/m²), mean	27.9 (27.8 to 28.0)
Maternal BMI category	
Underweight	0.9 (0.7 to 1.1)
Normal weight	28.9 (28.0 to 29.9)
Overweight	40.1 (39.1 to 41.1)
Obesity	30.2 (29.2 to 31.1)
Child haemoglobin (g/dL), mean	11.2 (11.2 to 11.3)
Anaemia severity in children	
Severe	0.1 (0.1 to 0.2)
Moderate	7.2 (6.8 to 7.7)
Mild	22.5 (21.8 to 23.3)
No anaemia	70.1 (69.3 to 71.0)
Child anaemia (dichotomous)	
No anaemia	70.1 (69.3 to 71.0)
Anaemia	29.9 (29.0 to 30.7)

95% CI: 95% confidence interval; BMI: body mass index.

The prevalence of anaemia was higher among children of mothers with normal weight (32.9%, p < 0.001) and among those with lower maternal BMI (27.5 kg/m^2^, p < 0.001). Other variables significantly associated with higher anaemia prevalence included younger child age (27.9 months, p < 0.001), younger maternal age (30.3 years, p < 0.001), rural area of residence (39.1%, p < 0.001), mothers who were married or cohabiting (30.3%, p = 0.020), residence in the highlands (37.3%, p < 0.001) or jungle region (38.1%, p < 0.001), higher altitude (1,379 metres above sea level, p < 0.001), no formal education (p < 0.001), lowest wealth index category (41.5%, p < 0.001), four or more children (35.6%, p < 0.001), unsafe or non-chlorinated drinking water (34.6%, p < 0.001), recent diarrhoea (35.6%, p < 0.001) or fever (34.9%, p < 0.001), and not receiving antiparasitic medication in last 12 months (32.7%, p < 0.001) (Table 2).

**Table 2:** Characteristics of children aged 6 to 59 months according to anaemia status (n = 16,973).

Variable	Child anaemia status		p-value
	No anaemia (70.1%) % or mean (95% CI)	Anaemia (29.9%) % or mean (95% CI)	
Child age (months), mean	34.1 (33.7 to 34.4)	27.9 (27.4 to 28.4)	<0.001
Maternal age (years), mean	31.4 (31.3 to 31.6)	30.3 (30.0 to 30.5)	<0.001
Child sex			0.073
Male	69.4 (68.2 to 70.6)	30.6 (29.4 to 31.8)	
Female	70.9 (69.7 to 72.1)	29.1 (27.9 to 30.3)	
Area of residence			<0.001
Urban	73.9 (72.9 to 74.9)	26.1 (25.1 to 27.1)	
Rural	60.9 (59.2 to 62.5)	39.1 (37.5 to 40.8)	
Marital status			0.020
Married/cohabiting	69.7 (68.7 to 70.6)	30.3 (29.4 to 31.3)	
Never married/not living together	72.1 (70.1 to 74.0)	27.9 (26.0 to 29.9)	
Widowed/divorced	82.3 (67.1 to 91.4)	17.7 (8.6 to 32.9)	
Natural region			<0.001
Metropolitan Lima	80.2 (78.1 to 82.1)	19.8 (17.9 to 21.9)	
Rest of the Coast	75.7 (74.3 to 77.1)	24.3 (22.9 to 25.8)	
Highlands	62.7 (61.1 to 64.4)	37.3 (35.6 to 38.9)	
Jungle	61.9 (60.1 to 63.6)	38.1 (36.4 to 39.9)	
Altitude (metres above sea level), mean	1026.0 (987.4 to 1064.7)	1379.5 (1322.6 to 1436.3)	<0.001
Educational level			<0.001
No formal education	57.7 (49.1 to 65.8)	42.3 (34.2 to 50.9)	
Primary	63.2 (60.9 to 65.5)	36.8 (34.5 to 39.1)	
Secondary	68.0 (66.8 to 69.3)	32.0 (30.8 to 33.2)	
Higher	76.3 (74.9 to 77.6)	23.8 (22.4 to 25.2)	
Wealth index			<0.001
Poorest	58.5 (56.9 to 60.2)	41.5 (39.8 to 43.2)	
Poor	69.1 (67.5 to 70.8)	30.9 (29.3 to 32.6)	
Middle	75.1 (73.3 to 76.9)	24.9 (23.1 to 26.7)	
Rich	78.9 (77.0 to 80.8)	21.1 (19.2 to 23.0)	
Richest	80.7 (78.2 to 83.0)	19.3 (17.0 to 21.9)	
Parity			<0.001
One child	73.1 (71.6 to 74.6)	26.9 (25.4 to 28.4)	
Two to three children	70.5 (69.4 to 71.6)	29.5 (28.4 to 30.6)	
Four or more children	64.4 (62.1 to 66.6)	35.6 (33.4 to 37.9)	
Water quality			<0.001
Safe water	75.5 (74.1 to 76.8)	24.6 (23.2 to 25.9)	
Partially chlorinated	70.7 (68.3 to 73.0)	29.3 (27.0 to 31.7)	
Unsafe/non-chlorinated	65.4 (64.0 to 66.7)	34.6 (33.3 to 36.0)	
Not measured	69.1 (66.5 to 71.6)	30.9 (28.4 to 33.5)	
Diarrhoea (past 2 weeks)			<0.001
No	71.0 (70.0 to 71.9)	29.0 (28.1 to 30.0)	
Yes	64.5 (62.0 to 66.8)	35.6 (33.2 to 38.0)	
Cough (past 2 weeks)			0.071
No	70.7 (69.6 to 71.7)	29.3 (28.3 to 30.4)	
Yes	69.1 (67.7 to 70.5)	30.9 (29.5 to 32.3)	
Fever (past 2 weeks)			<0.001
No	71.4 (70.5 to 72.3)	28.6 (27.7 to 29.5)	
Yes	65.1 (63.2 to 67.0)	34.9 (33.0 to 36.9)	
Antiparasitic medication (past 12 months)			<0.001
No	67.3 (66.2 to 68.5)	32.7 (31.5 to 33.8)	
Yes	74.3 (73.1 to 75.5)	25.7 (24.5 to 26.9)	
Maternal BMI (kg/m²), mean	28.0 (27.9 to 28.2)	27.5 (27.3 to 27.7)	<0.001
Maternal BMI category			<0.001
Underweight	70.2 (61.9 to 77.4)	29.8 (22.6 to 38.1)	
Normal weight	67.1 (65.4 to 68.7)	32.9 (31.3 to 34.6)	
Overweight	70.6 (69.2 to 71.9)	29.5 (28.1 to 30.8)	
Obesity	72.5 (71.0 to 74.0)	27.5 (26.0 to 29.0)	

95% CI: 95% confidence interval; BMI: body mass index.

**Fig 2:**
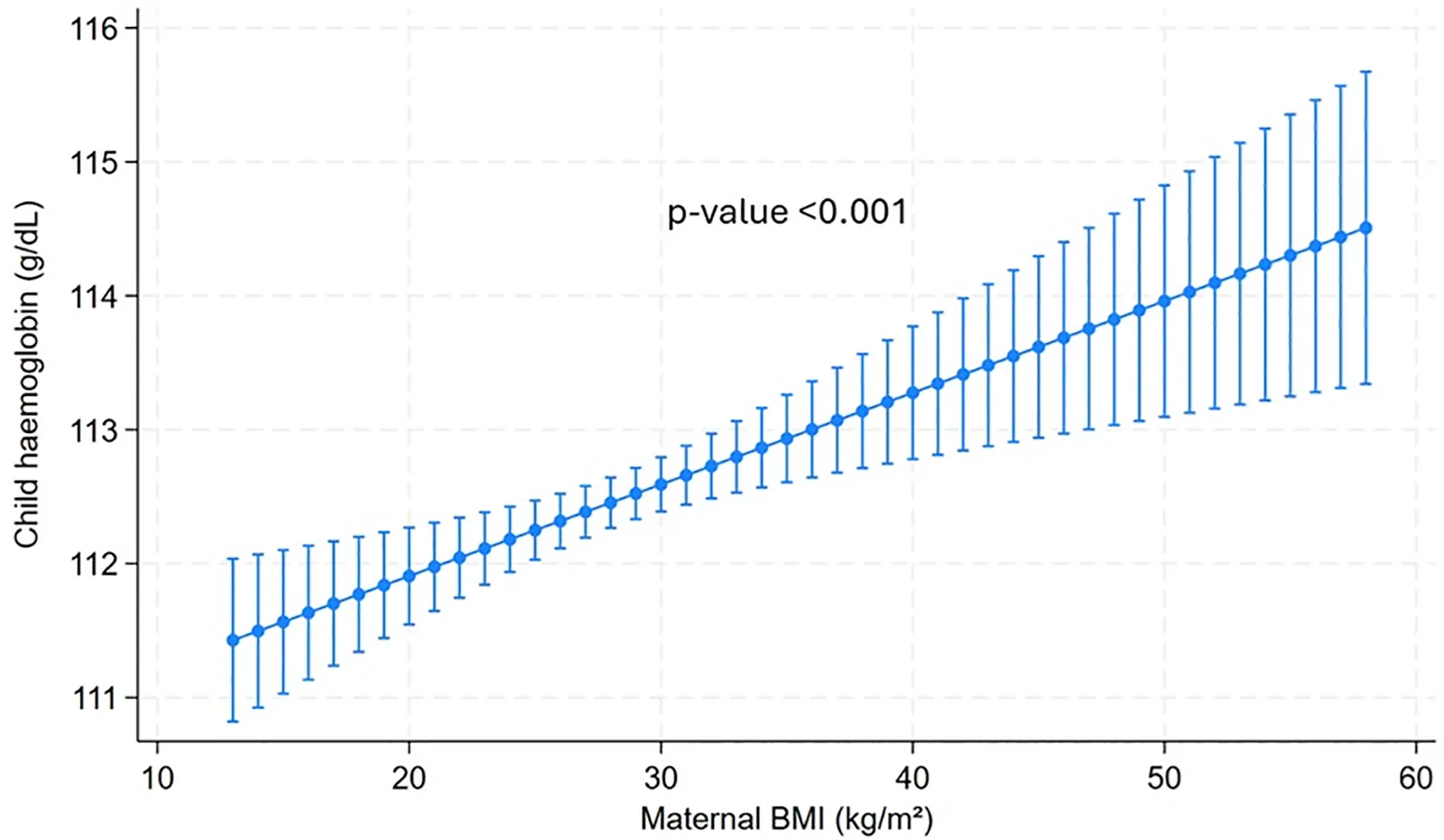
Association between maternal BMI and child haemoglobin levels.

When analysing the association between maternal BMI and anaemia prevalence in children, no significant association was observed in the adjusted analysis, either when BMI was evaluated categorically or as a continuous variable (Table 3). In contrast, children of mothers with obesity had haemoglobin levels 0.72 g/dL higher compared with those of mothers with normal weight (adjusted β 0.72; 95% CI: 0.23 to 1.22; p = 0.004). Furthermore, each 1 kg/m^2^ increase in maternal BMI was associated with an average increase of 0.07 g/dL in child haemoglobin levels (adjusted β 0.07; 95% CI: 0.03 to 0.11; p < 0.001) (Table 4). Finally, graphical representation showed a directly proportional and statistically significant relationship between maternal BMI and haemoglobin levels (Fig 2).

**Table 3:** Association between maternal BMI and anaemia prevalence in children aged 6-59 months (n = 16,973).

Variable	Crude PR (95% CI)	p-value	Adjusted PR* (95% CI)	p-value
Maternal BMI category				
Normal weight	Ref.		Ref.	
Underweight	0.90 (0.69 to 1.18)	0.460	0.90 (0.69 to 1.17)	0.442
Overweight	0.89 (0.84 to 0.96)	0.001	1.00 (0.94 to 1.07)	0.880
Obesity	0.83 (0.77 to 0.90)	<0.001	0.98 (0.91 to 1.06)	0.690
Maternal BMI (kg/m²)	0.984 (0.978 to 0.990)	<0.001	0.999 (0.991 to 1.002)	0.290

PR: prevalence ratio; 95% CI: 95% confidence interval; BMI: body mass index; Ref.: reference category.

*Adjusted for child age, maternal age (quartiles), child sex, area of residence, maternal marital status, natural region, altitude (quartiles), wealth index, parity, drinking water quality, history of diarrhoea, cough and fever in the past two weeks, and antiparasitic medication use in the past 12 months.

**Table 4:** Association between maternal BMI and child haemoglobin concentration (g/dL) among children aged 6-59 months (n = 16,973).

Variable	Crude β (95% CI)	p-value	Adjusted β* (95% CI)	p-value
Maternal BMI category				
Normal weight	Ref.		Ref.	
Underweight	−0.16 (−1.88 to 1.57)	0.859	0.40 (−1.23 to 2.03)	0.631
Overweight	1.52 (1.03 to 2.01)	<0.001	0.33 (−0.13 to 0.78)	0.156
Obesity	2.37 (1.84 to 2.90)	<0.001	0.72 (0.23 to 1.22)	0.004
Maternal BMI (kg/m²)	0.20 (0.16 to 0.24)	<0.001	0.07 (0.03 to 0.11)	<0.001

β: linear regression coefficient; 95% CI: 95% confidence interval; BMI: body mass index; Ref.: reference category.

*Adjusted for child age, maternal age (quartiles), child sex, area of residence, maternal marital status, natural region, altitude (quartiles), wealth index, parity, drinking water quality, history of diarrhoea, cough and fever in the past two weeks, and antiparasitic medication use in the past 12 months.

## Discussion

### Main findings

This cross-sectional analytical study conducted among children aged 6–59 months and their mothers in Peru found that maternal obesity was associated with higher haemoglobin levels in children. Furthermore, each 1 kg/m^2^ increase in maternal BMI was associated with an average increase of 0.07 g/dL in child haemoglobin, showing a linear relationship. However, no association was found between BMI and anaemia prevalence.

A previous cross-sectional study conducted in Nigeria among children aged 6–59 months found that maternal overweight (OR: 0.76; 95% CI: 0.61 to 0.96) and obesity (OR: 0.73; 95% CI: 0.57 to 0.94) were significantly associated with lower odds of childhood anaemia compared with underweight mothers [16]. Another cross-sectional study conducted among 96,804 children aged 6–59 months in Sub-Saharan Africa found that for each 1 kg/m^2^ increase in maternal BMI, child haemoglobin increased by 0.01 g/dL. Additionally, maternal underweight (OR: 1.20; 95% CI: 1.10 to 1.30) and normal weight (OR: 1.13; 95% CI: 1.07 to 1.19) were associated with higher odds of childhood anaemia compared with mothers with overweight or obesity [17]. Similarly, a study conducted in West Africa found that children of underweight mothers were more likely to have anaemia and underweight [18].

A plausible explanation for the positive association between maternal BMI and child haemoglobin is that maternal BMI may act as an indirect measure of household food security. Mothers with higher BMI may, on average, have greater access to foods rich in bioavailable iron and to preventive actions against anaemia. This is consistent with previous literature showing that higher maternal haemoglobin, child deworming and better household conditions were associated with higher child haemoglobin levels [17]. Another plausible mechanism is that mothers establish and maintain dietary patterns, food availability and feeding norms within the household, which may influence adequate micronutrient intake. In this sense, maternal BMI may reflect persistent dietary behaviours transmitted to the child during the critical complementary feeding period.

Another relevant finding was that, although a linear and statistically significant association was observed between maternal BMI and child haemoglobin levels, no significant differences were found when analysing anaemia as a dichotomous variable. This apparent discrepancy may be explained by the fact that, although the magnitude of the beta coefficient was greater than that reported in previous studies [17], the effect size was likely not large enough to shift a substantial number of children above the diagnostic cut-off point for anaemia. Thus, maternal BMI may exert a gradual influence on haemoglobin as a continuous parameter, but with a more limited impact on the clinical occurrence of anaemia defined categorically.

The directly proportional relationship reinforces the plausibility of a biological gradient between these variables. This finding may have population-level relevance, as even modest increases in haemoglobin levels may translate into substantial reductions in mortality among children under five years of age [3]. Additionally, the low proportion of underweight mothers in our sample (0.9%) limits statistical power to detect differences in this category, particularly considering that previous studies have identified a higher risk of anaemia at this extreme of BMI.

### Implications and recommendations

Because this study was based on a nationally representative survey with complex sampling, the findings are generalisable to Peruvian children aged 6–59 months and their non-pregnant mothers. The results suggest that interventions aimed at preventing and controlling childhood anaemia could benefit from a more comprehensive approach that includes maternal nutritional status and maternal dietary education. This does not imply promoting increases in BMI but rather improving diet quality and household feeding practices. Important knowledge gaps remain, including the lack of longitudinal studies to assess temporality of the association. Furthermore, the impact of maternal underweight in the Peruvian context remains unclear due to its low prevalence, which limits the precision of effect estimates. Future research could explore this relationship in specific subgroups and formally evaluate potential mediating mechanisms underlying the observed association.

### Limitations and strengths

This study has several limitations. First, its cross-sectional design precludes establishing temporality or inferring causality between maternal BMI and child haemoglobin levels or anaemia. Second, BMI was used as a proxy measure of maternal adiposity, which may underestimate or overestimate true body fat excess [25]. From an analytical perspective, although multiple relevant confounders were adjusted for, it was not possible to control for important factors such as iron supplementation, prematurity, type of breastfeeding due to a high proportion of missing data and child dietary diversity or feeding habits, which may result in residual confounding. Additionally, the low prevalence of underweight mothers limited the precision of estimates for this category. Nevertheless, the study also has important strengths. It used a nationally representative survey of Peru with complex sampling and an adequate sample size. Moreover, the association was evaluated using both anaemia as a dichotomous outcome and haemoglobin as a continuous variable (g/dL), allowing a more comprehensive understanding of the relationship and identifying a directly proportional association that reinforces its plausibility.

## Conclusion

Among Peruvian children aged 6–59 months, maternal obesity was associated with higher child haemoglobin levels, and each 1 kg/m^2^ increase in maternal BMI was associated with an average increase of 0.07 g/dL. However, maternal BMI was not associated with anaemia prevalence. These findings suggest a gradual effect on haemoglobin that does not necessarily translate into changes in anaemia defined by a diagnostic cut-off. Maternal nutritional status should be considered within comprehensive anaemia prevention strategies, and longitudinal studies are recommended to confirm these findings.
