# Supplementary material for: Maternal body mass index and child haemoglobin: A national cross-sectional study in Peru
Source: PLoS One. 2026 Sep 25;21(9):e0359236. doi: 10.1371/journal.pone.0359236 (PMC13614543; doi:10.1371/journal.pone.0359236)
Supplement: Supplementary file 1 [file pone.0359236.s001.pdf]

Methods:

- Variables: Please consider removing the database codes for the variables used, as these may not be necessary for the reader.
- Variables: Does this dataset include information on maternal, child, or household dietary diversity? If so, it would be important to include this covariate in the analyses.
- The use of VIF < 10 as a reference for assessing multicollinearity may not be the most appropriate choice. To strengthen the robustness of the analysis, it would be preferable to adopt a more conservative threshold, such as VIF < 5.
- It would also be important to assess whether area of residence, household wealth index, and drinking water quality are conceptually overlapping variables.
- Excluding variables with a high proportion of missing values may also lead to important losses in the Poisson regression analysis. Variables such as iron supplementation, type of breastfeeding, and prematurity are strong mediators of the serum iron levels evaluated in the article. For this reason, excluding these variables may introduce substantial confounding bias into the final results.
- It would therefore be helpful to report the proportion of missing values for these variables and to assess whether there is any systematic pattern related to the missingness. Finally, the authors should consider whether a data imputation strategy could be applied.
