# Supplementary material for: Maternal body mass index and child haemoglobin: A national cross-sectional study in Peru
Source: PLoS One. 2026 Sep 25;21(9):e0359236. doi: 10.1371/journal.pone.0359236 (PMC13614543; doi:10.1371/journal.pone.0359236)
Supplement: Supplementary file 2 [file pone.0359236.s003.docx]

**Response to Reviewers**

***Manuscript: “Maternal body mass index and child haemoglobin: A national cross-sectional study in Peru”***

We thank the editor and the reviewers for their careful reading of our manuscript and for their constructive comments, which helped us improve the clarity and rigour of several aspects of the work. Below, we address each comment individually.

**Reviewer 1**

**Comment 1.1.** *“On page 4, first paragraph, the citation for the anaemia classification in children aged 6–23 months is missing. If the same reference was used for older children, it should be cited the first time the classification is presented, at the end of the sentence referring to younger children.”*

**Response.** Thank you for pointing this out. The anaemia classification thresholds for both age groups were taken from the same source, the 2024 technical guidelines of the Peruvian Ministry of Health (reference 21). Following the reviewer’s suggestion, we have added this citation at the end of the sentence describing the classification for children aged 6–23 months, so that the reference now appears the first time the classification is presented.

**Changes in the Manuscript – Methods, Variables section, first paragraph (dependent variable):**

***Modified:*** *“Among children aged 6–23 months, severe anaemia was defined as haemoglobin <7.0 g/dL, moderate as 7.0–9.4 g/dL and mild as 9.5–10.4 g/dL [21]. Among children aged 24–59 months, severe anaemia was defined as <7.0 g/dL, moderate as 7.0–9.9 g/dL and mild as 10.0–10.9 g/dL [21].”*

**Reviewer 2**

**Comment 2.1.** *“Please consider removing the database codes for the variables used, as these may not be necessary for the reader.”*

**Response.** We agree. The database variable codes have been removed from the main text, since they are not essential for the reader. The variables remain fully identifiable by their names, and their questionnaire source is still indicated.

**Changes in the Manuscript – Methods, Variables section, first paragraph (dependent variable):**

***Modified:*** *“Altitude-adjusted haemoglobin levels in g/dL and anaemia status according to the updated 2024 technical guidelines of the Peruvian Ministry of Health and the World Health Organization (WHO) were used.”*

**Changes in the Manuscript – Methods, Variables section, second paragraph (independent variable):**

***Modified:*** *“The independent variable was BMI among women of reproductive age, obtained from the Individual questionnaire.”*

**Comment 2.2.** *“Does the dataset include information on maternal, child, or household dietary diversity? If so, it would be important to include this covariate in the analyses.”*

**Response.** Thank you for this observation. We reviewed the ENDES 2024 database and confirmed that it does not contain information on dietary diversity or feeding habits applicable to the study population (children aged 6–59 months). Breastfeeding is recorded only as exclusive breastfeeding up to six months of age, and the available indicators of fruit and vegetable consumption are restricted to individuals aged 15 years and older. Therefore, a dietary diversity covariate could not be constructed for the analysed sample. We have acknowledged this as a limitation in the revised manuscript.

**Changes in the Manuscript – Discussion, Limitations and strengths subsection, first paragraph (sentence added):**

***Modified:*** *“From an analytical perspective, although multiple relevant confounders were adjusted for, it was not possible to control for important factors such as iron supplementation, prematurity, type of breastfeeding due to a high proportion of missing data and child dietary diversity or feeding habits, which may result in residual confounding.”*

**Comment 2.3.** *“The use of VIF < 10 as a reference for assessing multicollinearity may not be the most appropriate choice. To strengthen the robustness of the analysis, it would be preferable to adopt a more conservative threshold, such as VIF < 5.”*

**Response.** We appreciate this suggestion. We chose to retain the VIF > 10 threshold because it is the conventional and most widely used criterion for detecting multicollinearity and corresponds to the rule of thumb explicitly documented in the statistical software used for our analysis. The Stata manual ([R] regress postestimation, estat vif) states that “most analysts rely on informal rules of thumb applied to the VIF” and that there is evidence of multicollinearity when “the largest VIF is greater than 10 (some choose a more conservative threshold value of 30)”, citing Chatterjee and Hadi (2012). The VIF < 10 criterion is therefore not a lenient choice but the standard reference implemented in the software and in the applied literature. In our models, all VIF values were well below this threshold (highest VIF = 6.61; mean VIF = 2.02), indicating no meaningful multicollinearity.

**Comment 2.4.** *“It would also be important to assess whether area of residence, household wealth index, and drinking water quality are conceptually overlapping variables.”*

**Response.** We appreciate this observation. We assessed whether area of residence, household wealth index and drinking water quality were conceptually or statistically redundant. Although these variables are related, as they all reflect underlying social determinants of health, they capture conceptually distinct dimensions: area of residence reflects the geographic context (urban/rural), the wealth index reflects household socioeconomic position, and drinking water quality reflects environmental and sanitary conditions. These dimensions are conceptually distinct and not interchangeable; for example, residing in a given area does not determine a household’s wealth, and a higher wealth level does not necessarily guarantee access to good-quality drinking water. This conceptual distinction was consistent with the collinearity assessment, in which no evidence of multicollinearity was observed among these variables. We therefore retained the three variables in the adjusted model.

**Comment 2.5.** *“Excluding variables with a high proportion of missing values may lead to important losses in the Poisson regression analysis. Variables such as iron supplementation, type of breastfeeding, and prematurity are considered important factors related to the outcome, and excluding them may introduce bias into the final results.”*

**Response.** Thank you for this comment. Missing data were handled following a pre-specified strategy based on the proportion of missing values for each variable: variables with less than 10% missing data were retained in the analysis; variables with 10–30% missing data were considered for multiple imputation; and variables with more than 30% missing data were excluded. Iron supplementation, type of breastfeeding and prematurity showed a high proportion of missing values (>30%), largely because ENDES collects them only for specific subgroups of children. Retaining them would have substantially reduced the analytic sample, and, given the magnitude of missingness, imputation would have relied on strong, unverifiable assumptions. We therefore excluded these variables and acknowledged the resulting potential for residual confounding as a limitation (Discussion, Limitations and strengths subsection). We have also made the missing-data strategy explicit in the Methods.

**Changes in the Manuscript – Methods, Statistical analysis section:**

***Modified:*** *“Missing data were handled according to a pre-specified strategy based on the proportion of missing values for each variable: variables with less than 10% missing data were retained in the analysis, those with 10–30% missing data were considered for multiple imputation, and those with more than 30% missing data were excluded. None of the variables met the criteria for multiple imputation; therefore, no multiple imputation was performed. Accordingly, variables related to iron supplementation, type of breastfeeding received and prematurity had a high proportion of missing values (61.9%, 41.9%, and 91.9%, respectively) and were therefore not included in the analysis.”*

**Comment 2.6.** *“Please report the proportion of missing values for iron supplementation, type of breastfeeding, and prematurity, and assess whether there is any systematic pattern related to the missingness.”*

**Response**. Thank you for this request. We now report the proportion of missing values for these three variables: iron supplementation 61.9%, type of breastfeeding 41.9%, and prematurity 91.9%. We also examined the pattern of missingness. In ENDES, these items are recorded only for specific subgroups of children defined by the survey design (for example, birth-related items such as prematurity for recent births, type of breastfeeding for children currently breastfeeding, and iron supplementation within defined age ranges), which is consistent with a structured, non-random pattern of missingness rather than data missing completely at random.

**Changes in the Manuscript – Methods, Statistical analysis section:**

***Modified:*** *“Missing data were handled according to a pre-specified strategy based on the proportion of missing values for each variable: variables with less than 10% missing data were retained in the analysis, those with 10–30% missing data were considered for multiple imputation, and those with more than 30% missing data were excluded. None of the variables met the criteria for multiple imputation; therefore, no multiple imputation was performed. Accordingly, variables related to iron supplementation, type of breastfeeding received and prematurity had a high proportion of missing values (61.9%, 41.9%, and 91.9%, respectively) and were therefore not included in the analysis.”*

**Comment 2.7.** *“The authors should consider whether a data imputation strategy could be applied for variables with substantial missing data.”*

**Response.** Thank you for this suggestion. As described above (Comment 2.5), our pre-specified strategy reserved multiple imputation for variables with 10–30% missing data; however, no covariate fell within this range, so imputation was not required for the variables included in the model.

**Changes in the Manuscript – Methods, Statistical analysis section:**

***Modified:*** *“Missing data were handled according to a pre-specified strategy based on the proportion of missing values for each variable: variables with less than 10% missing data were retained in the analysis, those with 10–30% missing data were considered for multiple imputation, and those with more than 30% missing data were excluded. None of the variables met the criteria for multiple imputation; therefore, no multiple imputation was performed. Accordingly, variables related to iron supplementation, type of breastfeeding received and prematurity had a high proportion of missing values (61.9%, 41.9%, and 91.9%, respectively) and were therefore not included in the analysis.”*
