# Supplementary material for: Maternal body mass index and child haemoglobin: A national cross-sectional study in Peru
Source: PLoS One. 2026 Sep 25;21(9):e0359236. doi: 10.1371/journal.pone.0359236 (PMC13614543; doi:10.1371/journal.pone.0359236)
Supplement: Peer review history

# Peer review: Decision Letter 0

**DOI:** 10.1371/journal.pone.0359236.r001

**Authors:** Marly Cardoso

9 Aug 2026

Dear Dr. Soriano-Moreno,

plosone@plos.org. When you're ready to submit your revision, log on to https://www.editorialmanager.com/pone/ and select the 'Submissions Needing Revision' folder to locate your manuscript file.

- A letter that responds to each point raised by the academic editor and reviewer(s). You should upload this letter as a separate file labeled 'Response to Reviewers'.
- A marked-up copy of your manuscript that highlights changes made to the original version. You should upload this as a separate file labeled 'Revised Manuscript with Track Changes'.
- An unmarked version of your revised paper without tracked changes. You should upload this as a separate file labeled 'Manuscript'.

If you would like to make changes to your financial disclosure, please include your updated statement in your cover letter. Guidelines for resubmitting your figure files are available below the reviewer comments at the end of this letter.

If applicable, we recommend that you deposit your laboratory protocols in protocols.io to enhance the reproducibility of your results. Protocols.io assigns your protocol its own identifier (DOI) so that it can be cited independently in the future. For instructions see: https://journals.plos.org/plosone/s/submission-guidelines#loc-laboratory-protocols. Additionally, PLOS ONE offers an option for publishing peer-reviewed Lab Protocol articles, which describe protocols hosted on protocols.io. Read more information on sharing protocols at https://plos.org/protocols?utm_medium=editorial-email&utm_source=authorletters&utm_campaign=protocols.

As the corresponding author, your ORCID iD is verified in the submission system and will appear in the published article. PLOS supports the use of ORCID, and we encourage all coauthors to register for an ORCID iD and use it as well. Please encourage your coauthors to verify their ORCID iD within the submission system before final acceptance, as unverified ORCID iDs will not appear in the published article. *Only*  the individual author can complete the verification step; PLOS staff *cannot*  verify ORCID iDs on behalf of authors.

We look forward to receiving your revised manuscript.

Kind regards,

Marly A. Cardoso, Ph.D.

Academic Editor

PLOS One

Journal Requirements:

When submitting your revision, we need you to address these additional requirements.

1. Please ensure that your manuscript meets PLOS ONE's style requirements, including those for file naming. The PLOS ONE style templates can be found at

https://journals.plos.org/plosone/s/file?id=wjVg/PLOSOne_formatting_sample_main_body.pdf and

https://journals.plos.org/plosone/s/file?id=ba62/PLOSOne_formatting_sample_title_authors_affiliations.pdf

2. If the reviewer comments include a recommendation to cite specific previously published works, please review and evaluate these publications to determine whether they are relevant and should be cited. There is no requirement to cite these works unless the editor has indicated otherwise.

3. Please review your reference list to ensure that it is complete and correct. If you have cited papers that have been retracted, please include the rationale for doing so in the manuscript text, or remove these references and replace them with relevant current references. Any changes to the reference list should be mentioned in the rebuttal letter that accompanies your revised manuscript. If you need to cite a retracted article, indicate the article’s retracted status in the References list and also include a citation and full reference for the retraction notice.

[Note: HTML markup is below. Please do not edit.]

Reviewers' comments:

Reviewer's Responses to Questions

**Comments to the Author**

1. Is the manuscript technically sound, and do the data support the conclusions?

Reviewer #1: Yes

Reviewer #2: Partly

**********

2. Has the statistical analysis been performed appropriately and rigorously?

Reviewer #1: Yes

Reviewer #2: Yes

**********

3. Have the authors made all data underlying the findings in their manuscript fully available?

The PLOS Data policy

Reviewer #1: Yes

Reviewer #2: Yes

**********

4. Is the manuscript presented in an intelligible fashion and written in standard English?

Reviewer #1: Yes

Reviewer #2: Yes

**********

Reviewer #1: The manuscript is well written and the findings supports earlier evidence linking maternal nutritional status with child's hemoglobin level.

Authors should note the following minor comment to improve manuscript:

On page 4, first paragraph: Citation for the anaemia classification of children 6-23 months is missing. If authors used the same cited for the older children they need to cite the first time used, i.e. at the end of sentence on younger children. this is in line with acceptable practice.

Reviewer #2: The manuscript draws on a highly qualified and appropriate data source for the analysis it aims to conduct. However, I would encourage the authors to carefully consider the methodological concerns raised above in order to strengthen the robustness of the analyses and the conclusions presented.

**********

what does this mean?). If published, this will include your full peer review and any attached files.

If you choose “no”, your identity will remain anonymous but your review may still be made public.

**Do you want your identity to be public for this peer review?**  For information about this choice, including consent withdrawal, please see our Privacy Policy

Reviewer #1: No

Reviewer #2: No

**********

[NOTE: If reviewer comments were submitted as an attachment file, they will be attached to this email and accessible via the submission site. Please log into your account, locate the manuscript record, and check for the action link "View Attachments". If this link does not appear, there are no attachment files.]

To ensure your figures meet our technical requirements, please review our figure guidelines: https://journals.plos.org/plosone/s/figures

You may also use PLOS’s free figure tool, NAAS, to help you prepare publication quality figures: https://journals.plos.org/plosone/s/figures#loc-tools-for-figure-preparation.

NAAS will assess whether your figures meet our technical requirements by comparing each figure against our figure specifications.

Attachment Submitted filename: Dual Burden of Malnutrition.pdf

# Author comment: Author response to Decision Letter 1

**DOI:** 10.1371/journal.pone.0359236.r002

15 Aug 2026

Response to Reviewers

Manuscript: “Maternal body mass index and child haemoglobin: A national cross-sectional study in Peru”

We thank the editor and the reviewers for their careful reading of our manuscript and for their constructive comments, which helped us improve the clarity and rigour of several aspects of the work. Below, we address each comment individually.

Reviewer 1

Comment 1.1. “On page 4, first paragraph, the citation for the anaemia classification in children aged 6–23 months is missing. If the same reference was used for older children, it should be cited the first time the classification is presented, at the end of the sentence referring to younger children.”

Response. Thank you for pointing this out. The anaemia classification thresholds for both age groups were taken from the same source, the 2024 technical guidelines of the Peruvian Ministry of Health (reference 21). Following the reviewer’s suggestion, we have added this citation at the end of the sentence describing the classification for children aged 6–23 months, so that the reference now appears the first time the classification is presented.

Changes in the Manuscript – Methods, Variables section, first paragraph (dependent variable):

Modified: “Among children aged 6–23 months, severe anaemia was defined as haemoglobin <7.0 g/dL, moderate as 7.0–9.4 g/dL and mild as 9.5–10.4 g/dL [21]. Among children aged 24–59 months, severe anaemia was defined as <7.0 g/dL, moderate as 7.0–9.9 g/dL and mild as 10.0–10.9 g/dL [21].”

Reviewer 2

Comment 2.1. “Please consider removing the database codes for the variables used, as these may not be necessary for the reader.”

Response. We agree. The database variable codes have been removed from the main text, since they are not essential for the reader. The variables remain fully identifiable by their names, and their questionnaire source is still indicated.

Changes in the Manuscript – Methods, Variables section, first paragraph (dependent variable):

Modified: “Altitude-adjusted haemoglobin levels in g/dL and anaemia status according to the updated 2024 technical guidelines of the Peruvian Ministry of Health and the World Health Organization (WHO) were used.”

Changes in the Manuscript – Methods, Variables section, second paragraph (independent variable):

Modified: “The independent variable was BMI among women of reproductive age, obtained from the Individual questionnaire.”

Comment 2.2. “Does the dataset include information on maternal, child, or household dietary diversity? If so, it would be important to include this covariate in the analyses.”

Response. Thank you for this observation. We reviewed the ENDES 2024 database and confirmed that it does not contain information on dietary diversity or feeding habits applicable to the study population (children aged 6–59 months). Breastfeeding is recorded only as exclusive breastfeeding up to six months of age, and the available indicators of fruit and vegetable consumption are restricted to individuals aged 15 years and older. Therefore, a dietary diversity covariate could not be constructed for the analysed sample. We have acknowledged this as a limitation in the revised manuscript.

Changes in the Manuscript – Discussion, Limitations and strengths subsection, first paragraph (sentence added):

Modified: “From an analytical perspective, although multiple relevant confounders were adjusted for, it was not possible to control for important factors such as iron supplementation, prematurity, type of breastfeeding due to a high proportion of missing data and child dietary diversity or feeding habits, which may result in residual confounding.”

Comment 2.3. “The use of VIF < 10 as a reference for assessing multicollinearity may not be the most appropriate choice. To strengthen the robustness of the analysis, it would be preferable to adopt a more conservative threshold, such as VIF < 5.”

Response. We appreciate this suggestion. We chose to retain the VIF > 10 threshold because it is the conventional and most widely used criterion for detecting multicollinearity and corresponds to the rule of thumb explicitly documented in the statistical software used for our analysis. The Stata manual ([R] regress postestimation, estat vif) states that “most analysts rely on informal rules of thumb applied to the VIF” and that there is evidence of multicollinearity when “the largest VIF is greater than 10 (some choose a more conservative threshold value of 30)”, citing Chatterjee and Hadi (2012). The VIF < 10 criterion is therefore not a lenient choice but the standard reference implemented in the software and in the applied literature. In our models, all VIF values were well below this threshold (highest VIF = 6.61; mean VIF = 2.02), indicating no meaningful multicollinearity.

Comment 2.4. “It would also be important to assess whether area of residence, household wealth index, and drinking water quality are conceptually overlapping variables.”

Response. We appreciate this observation. We assessed whether area of residence, household wealth index and drinking water quality were conceptually or statistically redundant. Although these variables are related, as they all reflect underlying social determinants of health, they capture conceptually distinct dimensions: area of residence reflects the geographic context (urban/rural), the wealth index reflects household socioeconomic position, and drinking water quality reflects environmental and sanitary conditions. These dimensions are conceptually distinct and not interchangeable; for example, residing in a given area does not determine a household’s wealth, and a higher wealth level does not necessarily guarantee access to good-quality drinking water. This conceptual distinction was consistent with the collinearity assessment, in which no evidence of multicollinearity was observed among these variables. We therefore retained the three variables in the adjusted model.

Comment 2.5. “Excluding variables with a high proportion of missing values may lead to important losses in the Poisson regression analysis. Variables such as iron supplementation, type of breastfeeding, and prematurity are considered important factors related to the outcome, and excluding them may introduce bias into the final results.”

Response. Thank you for this comment. Missing data were handled following a pre-specified strategy based on the proportion of missing values for each variable: variables with less than 10% missing data were retained in the analysis; variables with 10–30% missing data were considered for multiple imputation; and variables with more than 30% missing data were excluded. Iron supplementation, type of breastfeeding and prematurity showed a high proportion of missing values (>30%), largely because ENDES collects them only for specific subgroups of children. Retaining them would have substantially reduced the analytic sample, and, given the magnitude of missingness, imputation would have relied on strong, unverifiable assumptions. We therefore excluded these variables and acknowledged the resulting potential for residual confounding as a limitation (Discussion, Limitations and strengths subsection). We have also made the missing-data strategy explicit in the Methods.

Changes in the Manuscript – Methods, Statistical analysis section:

Modified: “Missing data were handled according to a pre-specified strategy based on the proportion of missing values for each variable: variables with less than 10% missing data were retained in the analysis, those with 10–30% missing data were considered for multiple imputation, and those with more than 30% missing data were excluded. None of the variables met the criteria for multiple imputation; therefore, no multiple imputation was performed. Accordingly, variables related to iron supplementation, type of breastfeeding received and prematurity had a high proportion of missing values (61.9%, 41.9%, and 91.9%, respectively) and were therefore not included in the analysis.”

Comment 2.6. “Please report the proportion of missing values for iron supplementation, type of breastfeeding, and prematurity, and assess whether there is any systematic pattern related to the missingness.”

Response. Thank you for this request. We now report the proportion of missing values for these three variables: iron supplementation 61.9%, type of breastfeeding 41.9%, and prematurity 91.9%. We also examined the pattern of missingness. In ENDES, these items are recorded only for specific subgroups of children defined by the survey design (for example, birth-related items such as prematurity for recent births, type of breastfeeding for children currently breastfeeding, and iron supplementation within defined age ranges), which is consistent with a structured, non-random pattern of missingness rather than data missing completely at random.

Changes in the Manuscript – Methods, Statistical analysis section:

Modified: “Missing data were handled according to a pre-specified strategy based on the proportion of missing values for each variable: variables with less than 10% missing data were retained in the analysis, those with 10–30% missing data were considered for multiple imputation, and those with more than 30% missing data were excluded. None of the variables met the criteria for multiple imputation; therefore, no multiple imputation was performed. Accordingly, variables related to iron supplementation, type of breastfeeding received and prematurity had a high proportion of missing values (61.9%, 41.9%, and 91.9%, respectively) and were therefore not included in the analysis.”

Comment 2.7. “The authors should consider whether a data imputation strategy could be applied for variables with substantial missing data.”

Response. Thank you for this suggestion. As described above (Comment 2.5), our pre-specified strategy reserved multiple imputation for variables with 10–30% missing data; however, no covariate fell within this range, so imputation was not required for the variables included in the model.

Changes in the Manuscript – Methods, Statistical analysis section:

Modified: “Missing data were handled according to a pre-specified strategy based on the proportion of missing values for each variable: variables with less than 10% missing data were retained in the analysis, those with 10–30% missing data were considered for multiple imputation, and those with more than 30% missing data were excluded. None of the variables met the criteria for multiple imputation; therefore, no multiple imputation was performed. Accordingly, variables related to iron supplementation, type of breastfeeding received and prematurity had a high proportion of missing values (61.9%, 41.9%, and 91.9%, respectively) and were therefore not included in the analysis.”

Attachment Submitted filename: Response to Reviewers.docx

# Peer review: Decision Letter 1

**DOI:** 10.1371/journal.pone.0359236.r003

**Authors:** Marly Cardoso

10 Sep 2026

Maternal body mass index and child haemoglobin: A national cross-sectional study in Peru

PONE-D-26-16320R1

Dear Dr. Soriano-Moreno,

We’re pleased to inform you that your manuscript has been judged scientifically suitable for publication and will be formally accepted for publication once it meets all outstanding technical requirements.

Within one week, you’ll receive an e-mail detailing the required amendments. When these have been addressed, you’ll receive a formal acceptance letter and your manuscript will be scheduled for publication.

An invoice will be generated when your article is formally accepted. Please note, if your institution has a publishing partnership with PLOS and your article meets the relevant criteria, all or part of your publication costs will be covered. Please make sure your user information is up-to-date by logging into Editorial Manager at Editorial Manager® and clicking the ‘Update My Information' link at the top of the page. For questions related to billing, please contact billing support.

If your institution or institutions have a press office, please notify them about your upcoming paper to help maximize its impact. If they’ll be preparing press materials, please inform our press team as soon as possible -- no later than 48 hours after receiving the formal acceptance. Your manuscript will remain under strict press embargo until 2 pm Eastern Time on the date of publication. For more information, please contact onepress@plos.org.

Kind regards,

Marly A. Cardoso, Ph.D.

Academic Editor

PLOS One

Additional Editor Comments (optional):

All reviewers´ comments have been addressed accordingly.

Reviewer #2: All comments have been addressed

**********

2. Is the manuscript technically sound, and do the data support the conclusions?

Reviewer #2: Yes

**********

3. Has the statistical analysis been performed appropriately and rigorously?

Reviewer #2: Yes

**********

4. Have the authors made all data underlying the findings in their manuscript fully available?

The PLOS Data policy

Reviewer #2: Yes

**********

5. Is the manuscript presented in an intelligible fashion and written in standard English?

Reviewer #2: Yes

**********

Reviewer #2: (No Response)

**********

what does this mean?). If published, this will include your full peer review and any attached files.

If you choose “no”, your identity will remain anonymous but your review may still be made public.

**Do you want your identity to be public for this peer review?**  For information about this choice, including consent withdrawal, please see our Privacy Policy

Reviewer #2: No

**********

# Editor report: Acceptance letter

**DOI:** 10.1371/journal.pone.0359236.r004

**Authors:** Marly Cardoso

PONE-D-26-16320R1

PLOS One

Dear Dr. Soriano-Moreno,

I'm pleased to inform you that your manuscript has been deemed suitable for publication in PLOS One. Congratulations! Your manuscript is now being handed over to our production team.

At this stage, our production department will prepare your paper for publication. This includes ensuring the following:

* All references, tables, and figures are properly cited

* All relevant supporting information is included in the manuscript submission,

* There are no issues that prevent the paper from being properly typeset

You will receive further instructions from the production team, including instructions on how to review your proof when it is ready. Please keep in mind that we are working through a large volume of accepted articles, so please give us a few days to review your paper and let you know the next and final steps.

Lastly, if your institution or institutions have a press office, please let them know about your upcoming paper now to help maximize its impact. If they'll be preparing press materials, please inform our press team within the next 48 hours. Your manuscript will remain under strict press embargo until 2 pm Eastern Time on the date of publication. For more information, please contact onepress@plos.org.

You will receive an invoice from PLOS for your publication fee after your manuscript has reached the completed accept phase. If you receive an email requesting payment before acceptance or for any other service, this may be a phishing scheme. Learn how to identify phishing emails and protect your accounts at https://explore.plos.org/phishing.

If we can help with anything else, please email us at customercare@plos.org.

Thank you for submitting your work to PLOS One and supporting open access.

Kind regards,

PLOS One Editorial Office Staff

on behalf of

Dr. Marly A. Cardoso

Academic Editor

PLOS One
